# Systematic Bibliometric and Visualized Analysis of Research Hotspots and Trends on Autism Spectrum Disorder Neuroimaging

**DOI:** 10.1155/2022/3372217

**Published:** 2022-07-18

**Authors:** Yi Lu, Li Zhang, Xing-yang Wu, Fang-rong Fei, Hui Han

**Affiliations:** ^1^Department of Nursing, Xinxiang Medical University, Xinxiang, China; ^2^Department of Rehabilitation, Children's Hospital of Nanjing Medical University, Nanjing, China; ^3^Department of Medical Engineering, Xinxiang Medical University, Xinxiang, Henan, China; ^4^Zhejiang Provincial Center for Disease Control and Prevention, Hangzhou, China; ^5^Department of Nursing, First Affiliated Hospital of Huzhou University, Huzhou, China

## Abstract

**Background:**

Autism spectrum disorder (ASD) is a chronic developmental disability caused by differences in the brain. The gold standard for the diagnosis of this condition is based on behavioral science, but research on the application of neurological detection to diagnose the atypical nervous system of ASD is ongoing. ASD neuroimaging research involves the examination of the brain's structure, functional connections, and neurometabolic. However, limited medical resource and the unique heterogeneity of ASD have resulted in many challenges when neuroimaging is utilized.

**Objective:**

This bibliometric study is aimed at summarizing themes and trends in research on autism spectrum disorder neuroimaging and at proposing potential directions for future inquiry.

**Methods:**

Citations were downloaded from the Web of Science Core Collection database on neuroimaging published from January 1, 2012, to December 31, 2021. The retrieved information was analyzed using Bibliometric.com, CiteSpace.5.8. R3, and VOS viewer.

**Results:**

A total of 1,363 papers were published across 58 regions. The United States was the leading source of publications. The League of European Research Universities published the largest number of articles (171). Burst keywords from 2018 to 2021 include identification and network. The clusters of references that continued into 2020 included graph theory, functional connectivity, and classification, which represent key research topics.

**Conclusions:**

Imaging data is being used to identify neuro-network models with higher accuracy for ASD discrimination. Functional near-infrared imaging is advantageous compared to other neuroimaging. In the future, research on systematic and accurate computer-aided diagnosis technology should be encouraged. Moreover, the study of neuroimaging of ASD in different psychological and behavioral states can inspire new ideas about the diagnosis and intervention training of ASD and should be explored.

## 1. Introduction

Autism spectrum disorder (ASD) is a neurodevelopmental disorder that manifests itself in infancy. Its primary symptoms are social communication and communication disorders, as well as repetitive stereotyped behavior [1]. Owing to the limited availability of accurate prenatal screening tools, early diagnostic biomarkers, and effective treatment methods, the prevalence of ASD has been increasing annually [2]. The results of the survey and monitoring points of the American Autism and Developmental Disorder Monitoring Network (ADMN) published in 2020 and 2021 revealed that in 2016, the overall prevalence of ASD in four-year-old children was approximately one in 64, and there was one ASD child in every 59 for this age group in the United States in 2018 [3, 4]. Standardized assessment and diagnostic methods are the gold standard for clinical diagnosis of ASD [5–7]. Although there is no complete description of the etiology and pathogenesis of ASD, research on the application of neurological detection to describe the atypical nervous system of ASD is ongoing.

ASD neuroimaging research examines the brain from a variety of perspectives including brain structure, functional connections, and neurometabolic [8–10]. Structural magnetic resonance imaging (sMRI) is a technique for examining the anatomical structure of the brain. Several sMRI studies have proved that there are structural abnormalities in the frontal lobe, temporal lobe, hippocampus, amygdala, and striatum of ASD subjects [11–16]. Functional neuroimaging of ASD brains mainly includes functional magnetic resonance imaging (fMRI), magnetic resonance spectroscopy (MRS), positron emission tomography (PET), single positron emission computed tomography (SPECT), diffusion tensor imaging (DTI), and functional near-infrared spectroscopy (fNIRS). These techniques are frequently used to investigate brain functional connectivity, metabolite content, nerve receptor distribution, and brain activation. fMRI studies involve the measurement and analysis of the changes in the degree of oxygenation in the local cerebral blood flow of subjects during a task state or resting state [17–19]. fNIRS measures the hemodynamic characteristics of the cerebral cortex via near-infrared spectroscopy to detect neural activity, which is a promising method for the early identification of quantitative biomarkers in autism sites [20, 21]. MRS can detect molecular behavior abnormalities associated with ASD [22]. As a molecular nuclear medicine imaging technology, SPECT and PET facilitate the study of cerebral blood perfusion, glucose metabolism, and protein metabolism by detecting the distribution of different radioactive tracers [23, 24]. DTI is a magnetic resonance imaging (MRI) technique that can reflect the burden of nervous system diseases [25].

Neuroimaging studies of ASD involve a variety of imaging methods and subgroups. However, a bibliometric analysis of this field has not been performed. The purpose of this study is to summarize themes and trends in research on autism spectrum disorder neuroimaging and to propose potential directions for future inquiry. We used bibliometric methods to analyze scientific citation index (SCI) papers in the field. The data included the references of countries, regions, institutions, journals, research categories, keywords, and references. In addition, as the core of this study, we established a visual and unbiased method for exploring the areas of high research activity and the frontiers of ASD neuroimaging research. The research methods, distribution, and influence of these published works are discussed. The future development space and potential challenges of ASD neuroimaging are also discussed. This report can serve as a reference for doctors, neuroimaging experts, and researchers in this field.

## 2. Data Sources and Research Methods

### 2.1. Data Sources

**Figure 1:**
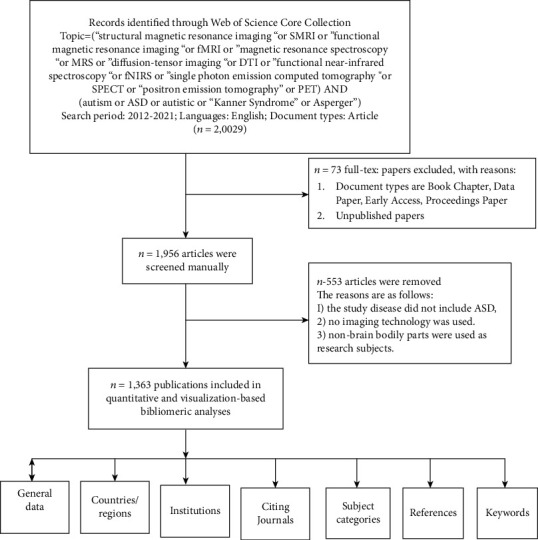
A frame flow diagram showing the detailed selection criteria and bibliometric analysis steps of neuroimaging of ASD.

The Web of Science Core Collection (WoSCC) database was chosen as the literature source for this study. Two authors (YL and WY) independently verified this work. The search formula was TS = (“structural magnetic resonance imaging” or sMRI or “functional magnetic resonance imaging” or fMRI or “magnetic resonance spectroscopy” or MRS or “diffusion-tensor imaging” or DTI or “functional near-infrared spectroscopy” or fNIRS or “single-photon emission computed tomography” or SPECT or “positron emission tomography” or PET) and (autism∗ or ASD or autistic or “Kanner Syndrome” or Asperger∗). The period was 2012–202, the language was English, and the document was published articles. We excluded book chapters, data papers, early access papers, and proceedings. The retrieval time was April 24, 2022. A total of 1956 English literatures were obtained. In addition, we manually screened the retrieved literature to avoid biased analysis results. Inclusion criteria are as follows: (1) research on ASD neuroimaging and (2) the research object can be human or animal. Exclusion criteria are as follows: (1) the study disease did not include ASD, (2) no imaging technology was used, and (3) the study sites were nonbrain body parts. Finally, the Web of Science (WoS) literature output function and the CiteSpace deduplication algorithm yielded 1363 effective literature. The detailed search and analysis processes are depicted in Figure 1.

## 3. Research Methods

Using the WoS data analysis module in CiteSpace5.R3 to visually analyze the collected data, the number of articles published each year, the country of origin, publishing organization, study categories, keywords, and references were all used to objectively assess the research status of ASD neuroimaging. The materials and methods section should contain sufficient detail so that all procedures can be repeated. It may be divided into headed subsections if several methods are described. https://bibliometric.com/app was used to show the volume of documents and the cooperation between countries.

## 4. Results and Discussion

### 4.1. Distribution of Articles by Publication Year

**Figure 2:**
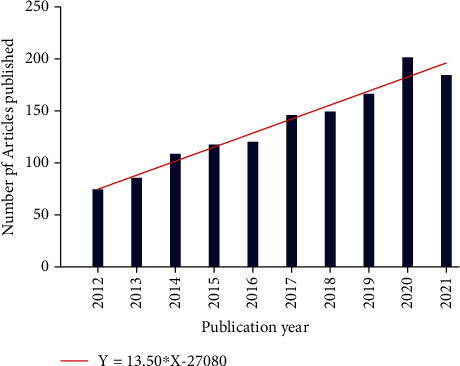
Trends in the number of publications on neuroimaging of ASD from 2012 to 2021.

To some extent, the number of articles published in academic journals on a particular topic reflects the level of interest in the research area. The annual publishing data, as well as their growth rate, can show the evolution of the field over time and the change in its level of importance.  Figure 2 depicts an overall increasing trend for the number of papers published from 2012 to 2021, with an average annual increase of approximately 14 articles. The popularity of ASD neuroimaging research is increasing. COVID-19 may have an impact on the number of papers published in 2021 compared to 2020. In addition, at present, some articles published in 2021 have not been included in the database.

### 4.2. Countries or Regions

**Figure 3:**
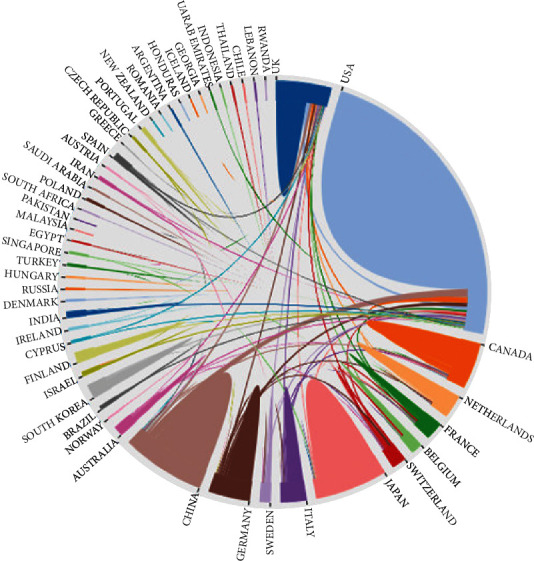
The cooperation of countries or regions that contributed to publications on neuroimaging of ASD from 2012 to 2021.

**Figure 4:**
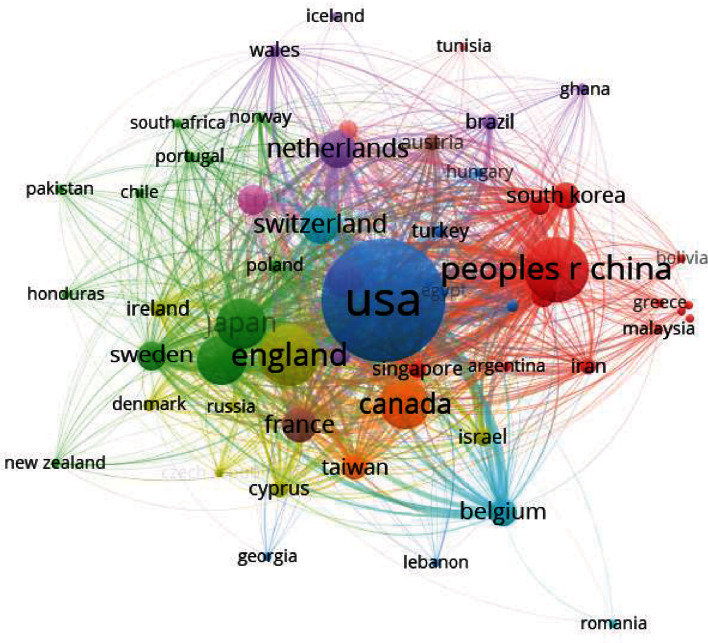
The cooperation of countries or regions that contributed to publications on neuroimaging of ASD from 2012 to 2021.

The 1363 publications on ASD neuroimaging included in the analysis originated from 58 nations or regions.  Figure 3 uses https://bibliometric.com/app to describe the number of documents submitted by each country and the cooperation between countries. The size of the areas with different colors indicates the number of documents submitted by the country represented by the label. The connection between regions indicates the existence of cooperative relationships among connected countries. For example, it is evident that the United States has the largest number of documents. Research that originated in the United States and the United Kingdom cooperates more closely with other countries.  Figure 4 shows the national cooperation network using the VOSviewer, which shows that the primary country in this field is the United States.  Table 1 quantifies the main findings. The default parameters of the CiteSpace program were used to generate centrality among them. The higher the centrality value, especially if it is larger than 0.1, the more essential the role. The United States (0.41), England (0.19), Germany (0.19), New Zealand (0.17), and the Netherlands (0.17) are the countries with the highest centrality among the top ten countries in terms of document volume (0.11). The *h*-index was derived from the WoS database search report and shows the article's influence. The two countries with the highest *h*-index are the United States (73) and the England (40). China has a large number of publications, but the centrality and *h*-index are small.

### 4.3. Institutions

The number and location of the top ten institutions are shown in Table 2. Their total number of documents accounts for 59.5% of all documents. Six American institutions are represented among these organizations: the University of California system (126 articles), Harvard University (86 articles), the University of North Carolina (55 articles), the University of Cambridge (53 articles), the University of North Carolina Chapel Hill (53 articles), and Yale University (51 articles). The number of documents issued by these institutions accounts for 52.28% of all documents issued by the top ten institutions. Furthermore, all of the top ten institutions are from developed countries.

### 4.4. Journals and Research Categories

**Figure 5:**
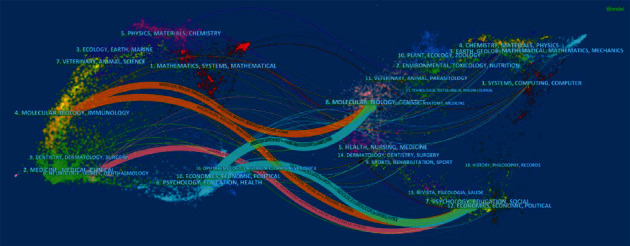
The dual map overlay of journals that contributed to publications on neuroimaging of ASD from 2012 to 2021.

We used CiteSpace to analyze citing journals and cited journals.  Figure 5 shows the citing and cited journals in different research fields. On the left is the research field of citing journals, which represents the research frontier. On the right is the research field of cited journals, which represents the knowledge base. The colored lines represent a research discipline for which research in a specific field is often cited. It is evident from the lines shown in Figure 5 that articles published in journals in the research field of molecular/biology/genetics are probably cited by journals in the research field of molecular/biology/immunology/psychology/education/health. Articles published in journals in the research field of psychology/education/social are usually cited by journals in the research field of molecular/biology/immunology/neurology/sports/ophthalmology. Tables 3 and 4 list the top ten citing journals and cited journals, respectively. It was determined that the knowledge base of ASD neuroimaging research is mostly neuroscience articles. Based on the research field of the citing journals, it is evident that genetics in neuroimaging of ASD has become a research frontier.

**Figure 6:**
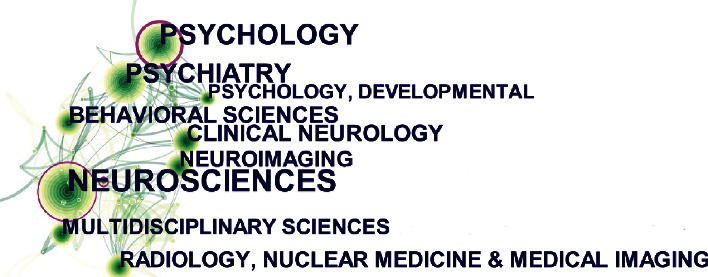
The network map of research categories for publications on neuroimaging of ASD from 2012 to 2021.

Figure 6 shows the top ten research categories with citations analyzed using the CiteSpace software. Each circular node represents a research category. The area generation size of the node table shows the number of research categories. The purple ring represents centrality, which identifies and measures the publication's importance. A node with a high centrality was considered to be a pivotal point in the publication [26]. The number of each study category was derived using the WoS citation analysis function and is shown in Table 5. Both Figure 6 and Table 5 show that neuroscience has the largest number of studies, whereas psychology is the most influential research category.

### 4.5. Keywords

**Figure 7:**
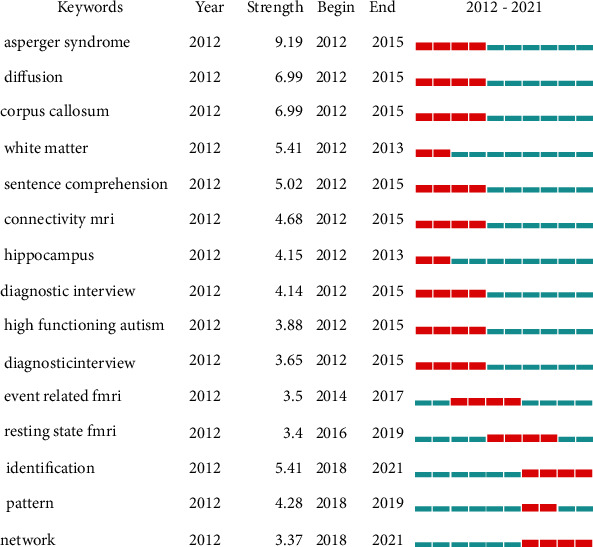
The keywords with the strongest citation bursts of publications on neuroimaging of ASD from 2012 to 2021.

CiteSpace was used to analyze the keywords that emerged over time, which is depicted in Figure 7. The red block represents the period of emerging keywords. Year per slice was set to 2. In order to extract the top 10% keywords in each time period, we set the top *n*% to 10%. And the minimum duration was set to 2. The keywords that emerged during the period from 2012 to 2015 were Asperger syndrome, diffusion, corpus callosum, white matter, sentence comprehension, connectivity MRI, hippocampus, diagnostic interview, and high functioning autism. The keywords that emerged from 2012 to 2017 were sense comprehension and task. From 2018 to 2020, the emergent keywords were event-related fMRI, resting-state fMRI, and pattern, whereas the emerging keywords from 2018 to 2021 included identification and network.

### 4.6. Citing Articles and References

Table 6 lists the 10 most frequently cited studies in the literature. fMRI was used in all these studies. The main challenges and limitations of these studies include the lack of description of the changes in global brain functional connectivity in ASD patients with age. The subjects were not representative, and there were instances of limited age groups and high-function autism. In addition, the selection of a control group is a challenging problem.

**Figure 8:**
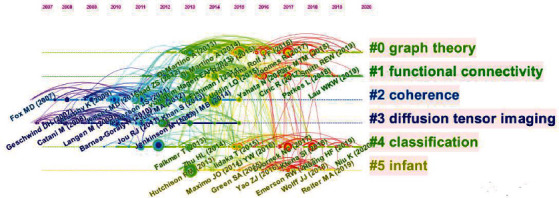
Cocited reference timeline map of publications on neuroimaging of ASD from 2012 to 2021.

The frequently cited literature had an important influence on their respective fields. We use the default setting of CiteSpace and a pruning algorithm to cluster the references. Indexing terms were used as the display of the clustering labels.  Figure 8 shows the active topics over time. The influence of the cluster to which the references belong is arranged from top to bottom. The cluster tags are “#0 graph theory,” “#1 functional connectivity,” “#2 coherence,” “#3 diffusion tensor imaging,” “#4 classification,” and “#5 infant.” The bold timeline in Figure 8 indicates the active topic. The popular reference clusters that lasted until 2015 include coherence and diffusion tensor imaging. The active topic from 2013 to 2019 is infant, and the actively investigated fields that remained popular until 2020 include graph theory and functional connectivity.

## 5. Discussion

### 5.1. Overall Results

It is evident from the above results that the number of documents exhibited an overall stable rising trend from 2012 to 2021. As shown in Figure 2, the average annual growth is 13.5 articles. The number of literature in 2021 was lower compared to 2020, probably because of the challenges introduced due to COVID-19 in terms of research on ASD [37].

In terms of the number of national documents, the United States ranks first in the number and centrality of the documents and has the highest *h*-index. This shows that this country is in a leading position in this research field. In addition, the research of developed countries such as England, Germany, Switzerland, and the Netherlands has a strong central position and influence. In terms of the distribution of documents, the top ten research institutions are concentrated in the United States, the United Kingdom, Canada, and several developed countries in Europe, which corresponds to the analysis results of national distribution. The number of articles published by the League of European Research Universities (LERU) ranks first and accounts for approximately 12.5% of the total number of studies. LERU is a University Alliance of 23 research universities located in 12 countries in Europe [38]. Based on its strong academic potential and professional knowledge, it has an important impact on European research, innovation, and higher education policies [39]. It is evident that the number of documents issued is related to the strength of interagency contacts. Further, when the institutions are more closely linked, researchers can obtain more research resources and more integration between various professional disciplines, promoting the growth of the field.

In addition to neuroimaging, it is evident from the research direction of the top ten cited journals that most of the articles involve behavioral science, neuroscience, psychiatry, psychology, and genetics. The top three research directions include neuroscience, psychiatry, and psychological development, which account for approximately 81% of the total. Therefore, we can see that ASD neuroimaging combines the research on psychological and behavioral development. In addition, the change in research hotspots can be estimated from the emerging keywords over time. From 2012 to 2017, the research focused on a certain brain region of high-function autism and the relationship between brain function and psychological and behavioral development. From 2018 to 2020, functional magnetic resonance imaging was used to perform brain imaging of autism in different states. From 2018 until recently, research on autism recognition and brain networks has been a research hotspot. The importance of magnetic resonance imaging in neuroimaging research of autism is evident based on the emerging keywords and top 10 citing articles. From the analysis of the cited literature, it is evident that the knowledge base of current research is also focused on the classification and brain function connection of ASD patients. From the research limitations and challenges of the top ten cited literatures, it can be concluded that the research still has limitations. The more prominent limitations are that the subjects are not representative enough, and the image signal is likely to be disturbed. Future studies need to include subjects of different ages and disease grades. Imaging techniques that are less susceptible to interference and appropriate for ASD subjects must be prioritized.

In general, research institutions should strengthen contacts and promote new research. Functional magnetic resonance imaging had a significant influence in the field of neuroimaging of ASD. In recent years, the research has focused on the identification of diseases and the functional connectivity of brain networks.

### 5.2. Research Hotspots

#### 5.2.1. Identification

At present, the gold standard for the diagnosis of ASD is based on behavioral science [40]. Research on the pathogenesis of ASD based on neuroimaging has been conducted, but this has not yet produced practical clinical applications [41].

One possible reason is that the recruited subjects are not fully representative of the ASD population in terms of symptoms and number [33, 35]. The complexity of brain connections and the heterogeneity of ASD hinder efforts to identify abnormal neurobiological signals [42, 43]. The combination of Autism Brain Imaging Data Exchange I (ABTDE I) and Autism Brain Imaging Data Exchange II (ABIDE II) provides researchers with a cross-sectional data set that allows for the selection of samples for scientific research [44]. Bi et al. proposed a cluster classifier with 100% accuracy to classify ASD patients and typical controls in the ABIDE database [45]. Dominic et al. used the four-dimensional resting-state fMRI obtained from ABIDE I to prove the detectability of ASD neuroimaging markers [46]. Eslami et al. designed a data enhancement strategy and used brain imaging data from 17 different brain imaging centers to generate the synthetic data set required to train a machine learning model to distinguish between the fMRI data of ASD. Finally, 82% classification accuracy was obtained [47]. Li et al. used ABIDE data set to evaluate a functional diagram discrimination network for ASD classification, which proved that this method can effectively distinguish between ASD patients and healthy controls [48].

In addition to the existing neuroimaging database, task state and resting-state ASD neuroimaging data can also identify atypical neurophysiological signals. Pretzsch et al. used fMRI analysis to determine that individuals with autism spectrum disorders have low functional connectivity (FC) between the ventral striatum and the frontal and pericentral regions (related to emotional, motor, and visual processing). In addition, they had higher striatum FC and higher putamen FC, and the temporal region involved speech and language [49]. A study conducted in Japan using fNIRS and a fragrance pulse injection system found that ASD participants with lower odor sensitivity presented reduced activity of the right dorsolateral prefrontal cortex in response to odor stimuli compared to a TD control group [50]. Several studies have also demonstrated that ASD has atypical features of FA and FC when performing language judgment, facial difference recognition, and memory tasks [51–53]. Neuroscience research on early identification of ASD or people at high risk for ASD has been emphasized [54, 55]. Earlier studies have established that the atypical development of ASD is related to the abnormal whole-brain connection, which may be congenital [56]. Padilla et al. studied the relationship between the MRI of infants with a gestational age of less than 27 weeks and preterm infants at full term and the future diagnosis of ASD (at the age of 6.5 years). It was determined that the MRI scan of ASD in infancy showed that the brain structure was different from that of typical development (TD) infants [57]. An fNIRS study found that compared to low-risk siblings of ASD subjects, high-risk siblings of ASD infants had reduced functional activity (FA) and FC during social interaction at 6 to 9 months, and hyperlinks were present between hemispheres at rest [58]. Many studies have shown that fNIRS is a promising tool to explore the neural development of ASD [59–61].

There are usually two ways to use neuroimaging for specific signal discrimination of ASD. One is to use public data sets, such as ABIDE, and the other is to analyze neural signal data under various task stimuli or resting states. A rigorous scientific evaluation of all task paradigms must be considered. Several studies have determined that fNIRS is a good choice for neural network connectivity analysis of ASD infants and young children compared to functional magnetic resonance imaging, because it has relatively high time resolution and is more tolerant to head motion [62, 63].

#### 5.2.2. Network

Neuroimaging technology can describe the neurophysiological patterns of different brain regions, which helps individuals to identify the abnormal brain structure and functional connections between TD individuals and nontypical development people.

Due to the heterogeneity of ASD, the description of atypical functional activation and functional connection of different brain regions in various states of ASD for a given period involves a variety of sensory organs or behavioral responses. For example, the theory-of-mental (ToM) network, including the medial prefrontal cortex, temporoparietal junction, inferior frontal gyrus, superior temporal sulcus, and posterior cingulate cortex, which is related to the development of key skills for effective social interaction, reflects the atypical ability of ASD subjects to emphasize and attribute views and intentions. Kana et al. demonstrated that when ASD individuals watched an animation of geometry, both FA and FC in the ToM network decreased [64]. Cao et al. found that the atypical development of the right temporoparietal junction may be a crucial aspect regarding the social defects of autistic patients from childhood to adolescence [65]. Farrant and Uddin found a hyperconnectivity of regions of interest in ASD children's attention network [66]. He et al. used MRI and discovered that the morphological connectivity of the cortical-striatum-thalamus-cortical network of ASD increased and the morphological connectivity of the cortical-cortical network decreased [16]. In addition, several studies have shown that the working memory neural network and visual-motor neural network of ASD exhibit different FC patterns compared to a TD group [67, 68].

In the field of medical imaging, artificial intelligence computer-aided diagnosis technology, including machine learning and deep learning algorithms, plays an important role [69]. The current research indicates that the development of new feature selection methods is an important direction of classification between ASD individuals and TD subjects, which can promote the application of machine learning methods to determine the most discriminative features [70]. A convolutional neural network (CNN) is an example of a deep learning algorithm, which can probably serve as a classifier for ASD recognition [71]. Wang et al. proposed a CCN architecture for fMRI analysis, which can effectively capture the FC characteristics of related applications in fMRI analysis [72]. A deep learning algorithm (deep belief network (DBN)) was used to combine the data from an Autism Brain Imaging Data Exchange I and II (ABIDE I and ABIDE II) to diagnose childhood ASD [73]. He et al. found that when the artificial neural network model is applied to the FC group data of very preterm infants, it can predict the cognitive results for 2 years old [74].

In general, the development of artificial intelligence-based models combined with neuroimaging datasets or task-based neurobiological signal data as a tool for diagnosing ASD has recently become an active area of research.

### 5.3. Study Limitations

The current research has some potential limitations. First, the downloaded citation only contains research in the WoSCC database for approximately a decade (2012-2021) and does not include earlier literature or that of other databases, which may cause a bias. Secondly, although the two authors manually removed irrelevant literature and participated in data analysis, this does not exclude subjective judgment. Third, some recent studies are in progress and have not been published, which will also bias the research results.

## 6. Conclusion

In conclusion, combining fMRI data to identify computer-aided diagnosis technology with higher accuracy for ASD discrimination and for determining the difference between FA and FC of neural networks that describe different states of ASD has become an area of active research. Among the countries involved in this research, the United States is the most influential in the field of ASD neuroimaging. Close cooperation between countries has positively impacted research progress. There are more fMRI-related studies than other neuroimaging studies. However, several studies are limited by their inadequate sample representation. To better address this problem, the strengthening of interagency contacts and the establishment of an ASD imaging database are recommended to promote scientific inquiry. In addition, fNIRS is proposed as a more suitable imaging research tool for the study of ASD neural FC and FA. In the future, research on systematic and accurate AI algorithms for ASD imaging data discrimination should be encouraged. This could serve as the basis for studies on neuroimaging of ASD in different psychological and behavioral states, which can inspire new ideas about the diagnosis and intervention training of ASD and should be explored. These findings can potentially inspire new ideas about the diagnosis and intervention training of ASD and serve as a reference for clinicians, rehabilitation trainers, and the staff of special education institutions.

## Figures and Tables

**Table 1:** The top 10 countries or regions with publications on neuroimaging of ASD from 2012 to 2021.

Rank	Countries or regions	Count	Centrality	*h*-index
1	USA	718	0.41	73
2	People's Republic of China	202	0.05	28
3	England	177	0.19	40
4	Japan	122	0.01	30
5	Canada	115	0.05	28
6	Germany	115	0.19	29
7	Italy	66	0.01	26
8	Netherlands	65	0.11	20
9	Switzerland	65	0.17	26
10	France	59	0.04	21

**Table 2:** The top 10 institutions with publications on neuroimaging of ASD from 2012 to 2021.

Rank	Institutions	Country	Count	% of 1,363
1	League of European Research Universities	Britain, Ireland, France, German, Italy, Sweden, Switzerland, Spain, Belgium, Finland, Denmark, Netherlands	171	12.546
2	University of California system	America	126	9.244
3	University of London	England	93	6.823
4	Harvard University	America	86	6.31
5	King's College London	England	68	4.989
6	University of North Carolina	America	55	4.035
7	University of Toronto	Canada	55	4.035
8	University of Cambridge	America	53	3.888
9	University of North Carolina Chapel Hill	America	53	3.888
10	Yale University	America	51	3.742

**Table 3:** The top 10 citing journals of publications on neuroimaging of ASD from 2012 to 2021.

Rank	Citing journals	Research fields	Count	2020 journal impact factor
1	Autism Research	Medicine/behavioural science	73	5.216
2	NeuroImage: Clinical	Medicine/neuroimaging	64	4.881
3	Frontiers in Human Neuroscience	Medicine/neuroscience	51	3.169
4	Human Brain Mapping	Medicine/neuroimaging	47	5.038
5	Journal of Autism and Developmental Disorders	Psychology, developmental	47	4.291
6	Cerebral Cortex	Medicine/neuroscience	44	5.357
7	Translational Psychiatry	Medicine/psychiatry	42	6.222
8	Molecular Autism	Medicine/genetics	40	7.509
9	Frontiers in Neuroscience	Medicine/neuroscience	39	3.59
10	PLOS One	Multidisciplinary science	37	3.24

**Table 4:** The top 10 cited journals of publications on neuroimaging of ASD from 2012 to 2021.

Rank	Cited journals	Research fields	Count	2020 journal impact factor
1	NeuroImage	Medicine/neuroscience	1203	6.556
2	Journal of Autism and Developmental Disorders	Psychology, developmental	1005	4.291
3	Biological Psychiatry	Medicine/neuroscience	902	13.382
4	Brain	Medicine/clinical neurology	892	13.501
5	Human Brain Mapping	Medicine/neuroimaging	892	5.038
6	Cerebral Cortex	Medicine/neuroscience	872	5.357
7	Journal of Neuroscience	Medicine/neuroscience	845	6.167
8	Proceedings of the National Academy of Sciences of the United States of America	Multidisciplinary sciences	835	11.2048
9	PLOS One	Multidisciplinary sciences	766	3.24
10	Neuron	Medicine/neuroscience	697	17.173

**Table 5:** The top 10 research categories for publications on neuroimaging of ASD from 2012 to 2021.

Rank	Research categories	Count	% of 1,363	Centrality
1	Neurosciences	1203	46.809	0.17
2	Psychiatry	1005	19.369	0.08
3	Psychology developmental	902	14.6	0.25
4	Neuroimaging	892	13.94	0.00
5	Behavioural sciences	892	9.244	0.01
6	Clinical neurology	872	8.437	0.04
7	Psychology	845	8.217	0.25
8	Radiology nuclear medicine medical imaging	835	7.924	0.03
9	Multidisciplinary science	766	6.163	0.00
10	Psychology experimental	697	6.016	0.00

**Table 6:** The top 10 citing articles on neuroimaging of ASD from 2012 to 2021.

Rank	Title of citing documents	DOI	Times cited	Imaging technology	Interpretation of the findings	Research limitations or challenges
1	“The Autism Brain Imaging Data Exchange: Towards a Large-Scale Evaluation of the Intrinsic Brain Architecture in Autism” [27]	10.1038/mp.2013.78	782	fMRI	This study found that the internal functional connectivity of the whole brain of ASD exhibited the coexistence of high connectivity and low connectivity. The dysfunction sites of ASD lie in the middle and rear of the insula, the posterior cingulate gyrus, the cortex, and the thalamus.	(1) It is necessary to study the dynamic changes in brain function and age development of autism (2) Researchers should focus on standardized phenotypes, including an extended diagnostic assessment, and comprehensively describe the brain-behavior relationship of dimensions (3) Physiological measurements that can index ASD related brain dysfunction should be considered
2	“Differences in White Matter Fiber Tract Development Present from 6 to 24 Months in Infants with Autism” [28]	10.1176/appi.ajp.2011.11091447	415	MRI	This research established that longitudinal data are essential for categorizing the dynamic age-related brain and behavior changes at the core of this neurodevelopmental disorder. In the first year of life, abnormal development of white matter pathways may precede the manifestation of autistic symptoms.	(1) In this study, only high-risk ASD siblings were included, and the absence of a low-risk control group limited the interpretation of results beyond ASD family background (2) Follow-up evaluation provides a positive guarantee for the diagnostic results
3	“Single Subject Prediction of Brain Disorders in Neuroimaging: Promises and Pitfalls” [29]	10.1016/j.neuroimage.2016.02.079	375	MRI	This study shows that neuroimaging data have great potential in predicting various diseases in a single subject. At present, the limited sample size is a problem, which can be solved by the modern data sharing model discussed in this paper.	(1) This review examined limited diseases (2) Some potential problems in the research were covered in this review, such as experimental design, the influence of head movement, and other factors on fMRI research
4	“Brain Hyperconnectivity in Children with Autism and Its Links to Social Deficits” [30]	10.1016/j.celrep.2013.10.001	293	fMRI	This study found that the brains of autistic patients are highly connected in function, leading to their social dysfunction.	ASD subjects do not fully represent the characteristics of this group
5	“Identification of Autism Spectrum Disorder Using Deep Learning and the ABIDE Dataset” [31]	10.1016/j.nicl.2017.08.017	253	fMRI	This study objectively identified the functional connection patterns of ASD participants from fMRI data.	This study failed to provide an overall assessment of autism classification. The use of resting-state fMRI data does not meet the biomarker criteria
6	“Deriving Reproducible Biomarkers from Multi-site Resting-State Data: An Autism-Based Example” [32]	10.1016/j.neuroimage.2016.09.038	241	fMRI	This study proved the feasibility of using fMRI to classify the neuropsychiatric states of autism.	(1) There is a deviation in the representativeness of the study samples (2) The predictive indicators of the classification model only use accuracy and do not use additional criteria, such as sensitivity and specificity (3) Limitations of data sets included in the study
7	“Altered Functional and Structural Brain Network Organization in Autism” [33]	10.1016/j.neuroimage.2016.10.045	240	fMRI	Children and adolescents with ASD demonstrated typical age-related modifications in the balance of local and global efficiency between structural and functional networks. And this imbalance was related to the severity of ASD individuals' socio-communicative deficiencies.	(1) This study was limited to ASD high functioning children and adolescents (2) More imaging acquisition, more adaptable modeling methods, and large-scale collaborative mechanism research will be encouraged (3) The comparison with other neurological diseases and the study of potential mechanisms such as genetic risk factors are very important for the description of brain network abnormalities in autism
8	“Fractionation of Social Brain Circuits in Autism Spectrum Disorders” [34]	10.1016/j.nicl.2012.11.006	213	fMRI	This study found reduced connectivity between social brain regions. In addition, the connections between the regions supporting language and sensory-motor processes and limbic-related brain regions were also selective.	(1) The subjects' ASD symptoms were underrepresented (2) Selecting an appropriate control group was a challenging task
9	“Impaired Thalamocortical Connectivity in Autism Spectrum Disorder: A Study of Functional and Anatomical Connectivity” [35]	10.1093/brain/aws160	200	fMRI and DTI	Compared with matched participants with normal development, the anatomical connectivity and functional connectivity of ASD children and adolescents were generally reduced.	(1) The subjects included in this study are underrepresented (2) The correlation between ASD thalamic connectivity index and the neuropsychological score is affected by variability (3) The specificity of the connection between the narrower specialized region and the thalamus, and any abnormality may not be found
10	“Default Mode Network in Childhood Autism: Posteromedial Cortex Heterogeneity and Relationship with Social Deficits” [36]	10.1016/j.biopsych.2012.12.013	197	fMRI	The precuneus showed hypoconnectivity with the visual cortex, basal ganglia, and locally within the posteromedial cortex in ASD children. The severity of social impairments in ASD was linked to abnormal posterior cingulate cortex hyperconnectivity, but precuneus hypoconnectivity was unrelated to social deficits.	(1) The age of the subjects selected in the study of the coordinate definition reference of the region of interest is different from that in this study (2) Potential impact of uncontrolled drugs and comorbid diseases on study results

## Data Availability

The underlying data used to support the findings of this study are available from the corresponding author upon request.
